# The attitude of Polish consumers toward food irradiation as one of the methods of food preservation

**DOI:** 10.3389/fpubh.2022.1047127

**Published:** 2023-01-05

**Authors:** Marta Buczkowska, Anna Dudczak, Dominika Szajnoga, Michał Górski, Jolanta Malinowska-Borowska, Aleksandra Kulik, Anna Szczyrba

**Affiliations:** ^1^Department of Chronic Diseases and Civilization-related Hazards, Faculty of Health Sciences in Bytom, Medical University of Silesia, Katowice, Poland; ^2^Second Scientific Circle of Department of Chronic Diseases and Civilization-related Hazards, Faculty of Health Sciences in Bytom, Medical University of Silesia, Katowice, Poland; ^3^Doctoral School of the Medical University of Silesia in Katowice, Faculty of Health Sciences in Bytom, Medical University of Silesia, Katowice, Poland

**Keywords:** food irradiation, preserved food, consumer attitude surveys, Polish consumers, change in consumer attitudes toward irradiation

## Abstract

**Background:**

Food irradiation is one of the methods of food preservation. Unfortunately, despite many opinions from national and international organizations that confirm the safety of the irradiation technique, the irradiated food market is slowly developing, which is particularly noticeable in European countries, including Poland.

**Objectives:**

The main objective of this study was to determine the attitude of Polish consumers toward irradiated food and to find out whether familiarizing the respondents with educational materials on the irradiation technique would change their attitudes.

**Material and methods:**

In response to the objective of the study, an online survey (with the presentation of educational materials) was conducted with 609 respondents living in the Silesian Voivodeship, Poland. A specially prepared author's questionnaire was used, containing questions relating to: sociodemographic data, food preservation, food irradiation. An integral part of the survey was a multimedia presentation containing information about the food irradiation process.

**Results:**

A low level of knowledge about food irradiation was found-−90.31% (*n* = 550) of the respondents had never heard of this method of preservation before. The percentage of respondents with a positive attitude toward radiation-preserved products increased significantly after providing informative material, from 6.20 (*n* = 38) to 67.16% (*n* = 409). The final attitude of the respondents toward irradiated food varied and depended on age, education and place of residence—positive attitudes toward irradiation predominated among those who were young (<30 years old), had a higher education and lived in cities >100,000 inhabitants. Educational materials also had a significant impact on consumers' interest in purchasing irradiated food—the percentage of people declaring a willingness to purchase this type of product increased from 19.20 (*n* = 117) to 59.30% (*n* = 361). Almost 60% of the respondents were willing to purchase irradiated foods. Women, on average, were more likely to be interested in purchasing irradiated food compared to men.

**Conclusions:**

The survey indicates that irradiated food could be commercially introduced in Poland, but on the condition that an effective educational program is planned.

## Introduction

According to current legislation, food irradiation is a technique which uses the ionizing radiation to preserve foodstuffs. Food irradiation is used to destroy microbial factors that are 52 responsible for food spoilage and/or harmful to consumers and plants. The use of ionizing radiation is also authorized to inhibit germination processes in agricultural produce. The disadvantages resulting from the potential harmfulness of irradiated food are not noted at lower radiation doses (<10 kGy), which is why higher doses are prohibited in the European Union (EU), including Poland (1–3). The safety of products irradiated with low (<1 kGy) and medium (1–10 kGy) doses has been confirmed by many recognized international organizations, including Food and Agriculture Organization (FAO of the United Nations) World Health Organization (WHO), European Food Safety Authority (EFSA), FDA (U.S. Food and Drug Administration), and The American Dietetic Association (ADA) (4, 5).

Not every food item can be treated with ionizing radiation, and the list of those which are allowed to be irradiated is regulated at the EU and national law level. According to the EU list, only one category of food products can be irradiated. These are dried aromatic herbs, spices, and vegetable seasonings. In contrast, Polish regulations allow additional irradiation of potatoes, onions, garlic, fresh and dried mushrooms, and dried vegetables. Currently, Poland does not commercially irradiate food (6, 7). Similar regulations are in force in other countries, hence the categories of food permitted for irradiation vary widely globally. For example, the FDA has approved the use of irradiation for beef, pork, shellfish, fresh fruits and vegetables, lettuce and spinach, poultry, sprouts, shell eggs, mollusks (including oysters, clams, scallops), and spices (8).

The market for irradiated food varies from one region of the world to another, not only in terms of the type of products but also in terms of their quantity. In Europe, <4,000 tons of radiation-preserved foods were marketed in 2019, which is a decrease of about 57% compared to the record year 2010 (9). At the same time, irradiation is becoming increasingly popular in many countries around the world, especially the United States or Asian countries, and this method has made it possible to minimize the phytosanitary risks associated with international transport. According to Eustice (10), in 2015, 23 million tons of irradiated food were commercially traded in the USA, 600,000 tons in China, 24,000 tons in South Africa, compared to only 5,700 tons in all countries of the European Union.

Although more than 100 years have passed since the first food application of ionizing radiation, the technique is still a little-known method of food preservation, especially in most European countries. The reason for this is the often observed reluctance of consumers to buy radiation-preserved items, and proper education seems to be the key to acceptance. Consumer research clearly shows that, given the choice and access to basic but reliable information about radiation, consumers are not only willing to buy irradiated foods, but often prefer them to conventionally preserved foods. Therefore, it is very important to carry out activities to increase consumer knowledge of food irradiation (11).

The main objective of this study was to determine the attitude of Polish consumers toward irradiated food and to find out whether familiarizing respondents with educational materials on the irradiation technique would change their attitude. The additional objective of the study was to determine the attitude of the respondent toward preserved foods, as irradiation is one of the methods of preserving food products.

The present study is of particular importance due to the lack of consumer research on a similar topic in Poland and few scientific reports from other European countries.

## Materials and methods

The study was survey-based and was conducted between November 2020 and February 2021. A specially prepared author's questionnaire was used, containing questions relating to:

- sociodemographic data (gender, age, place of residence, and education);
- food preservation (attitudes toward buying preserved food; advantages of food preservation; frequency of buying foods treated with different methods);
- food irradiation (identification of the irradiated food symbol; attitudes toward and purchase options for irradiated food; the importance of the advantages of irradiated food).

A multimedia presentation containing the following information was an integral part of the survey:

- WHO data on the prevalence of food-related diseases and their complications around the world (12);
- purposes of food irradiation, effects of the process on nutrients, and process advantages (2, 8, 13);
- photographs showing irradiated and unirradiated food products after a certain storage time (2).

Questions about irradiated food were made available to respondents before and after reading the educational materials.

Participation in the survey was voluntary and anonymous. Due to the epidemic situation, the survey was conducted online using Google Forms and was shared on social networks.

The survey used a random (simple) sampling method. The survey was conducted among the inhabitants of the Silesian Voivodeship, whose population represents 12% (4,492,000 persons in 2020) of the population of Poland (38,265,000 persons in 2020) (14, 15). In determining the minimum sample size, the following formula was adopted (16):


(1) $$\begin{array}{l} Nmin=\frac{Np\left(\alpha^{2}\times f\left(1-f\right)\right)}{Np\;\times {\;e}^{2}+\alpha^{2}\;\times \;f\left(1-f\right)} \end{array}$$

where: *Nmin*, minimum sample size; *Np*, population size from which the sample is taken; α, the adopted confidence level for the results (confidence level assumed = 95%); *f*, assumed fraction size – assumed unknown fraction (50%)*; e*, assumed maximum error, expressed as a fraction. Assumed at the level of 0.05.

According to the above formula, the minimum representative number of respondents (*Nmin*) has been determined at 384, but due to the lack of similar surveys in Poland, it was decided to increase the study group. Finally, it was possible to obtain 609 correctly completed questionnaires.

Statistical analysis was performed using Statistica 13.3 PL software (StatSoft Polska, Krakow, Poland). Tests for significance of differences were used: chi^2^ (Chi2), Mann–Whitney *U* (M–W), Kruskal–Wallis with *post-hoc* test (K–W), Bhapkar (B), Friedman (F), and Wilcoxon (W). The V-Cramer correlation coefficient (*V*) was used in the analysis of associations between the study variables. Results for which *P* < 0.05 were considered statistically significant.

## Results

### Characteristics of the study group

Of the 609 respondents, 72.60% (*n* = 442) were female and 27.40% (*n* = 167) were male. The mean age was 25.2 ± 8.5 years. Concerning age categories, respondents in the range 21–30 years (*n* = 374; 61.4%) constituted the largest group (*P* < 0.05).

In the study group, almost 90% of the respondents had a university degree (*n* = 271; 44.50%) or secondary/professional education (*n* = 276; 45.3%). The respondents' place of residence was most often a city of more than 100,000 inhabitants (*n* = 306; 50.10%) and a village (*n* = 148; 24.30%). Detailed characteristics of the study group by gender are presented in Table 1.

**Table 1:** Characteristics of the study group by gender.

	**Total *n* (%)**	**Gender**		***P*** **_chi2_ value**
		**Female *n* (%) 442 (72.60)**	**Male *n* (%) 167 (27.40)**	
**Age group**				
18–20	139 (22.80)	109 (24.70)	30 (18.00)	0.023
21–30	374 (61.40)	276 (62.40)	98 (58.60)	
31–40	48 (7.90)	29 (6.60)	19 (11.40)	
41–50	35 (5.80)	21 (4.70)	14 (8.40)	
≥51	13 (2.10)	7 (1.60)	6 (3.60)	
**Educational level**				
Primary education	62 (10.20)	46 (10.40)	16 (9.60)	0.51
Secondary/professional education	276 (45.30)	194 (43.90)	82 (49.10)	
Higer education	271 (44.50)	202 (45.70)	69 (41.30)	
**Place of residence**				
Village	148 (24.30)	111 (25.10)	37 (22.20)	0.65
City, with populations < 50,000	87 (14.30)	61 (13.80)	26 (15.50)	
City, with populations 50,000–100,000	69 (11.30)	53 (12.00)	16 (9.60)	
City, with populations >100,000	305 (50.10)	271 (49.10)	88 (52.70)	

P_chi2_–ch^2^ test.

### The attitude of the respondents toward food preservation

Almost 90% of respondents (*n* = 535; 87.90%) declared that they buy preserved food items, which only half (*n* = 346; 56.80%) paid attention to the method of preservation used. The willingness of the respondents to purchease preserved food was influenced by selected sociodemographic factors, such as gender, age, and education—the proportion of respondents who declared that they would buy preserved foods was highest among women (90.72%), respondents aged 21–30 years (92.78%) and those with higher education (91.51%; Table 2).

**Table 2:** Declaration of purchase of preserved food by sociodemographic factors.

	**Declaration of purchase of preserved food**		***P*** **_chi2_ value**
	**Yes *n* (%) 535 (87.85)**	**No *n* (%) 74 (12.15)**	
**Gender**			
Female	401 (90.72)	41 (9.28)	<0.001
Male	134 (80.24)	33 (19.76)	
**Age group**			
18–20	118 (84.89)	21 (15.11)	<0.001
21–30	347 (92.78)	27 (7.22)	
31–40	37 (77.08)	11 (22.92)	
41–50	25 (71.43)	10 (28.57)	
≥51	8 (61.54)	5 (38.46)	
**Educational level**			
Primary education	48 (77.42)	14 (22.58)	0.006
Secondary/professional education	239 (86.59)	37 (13.41)	
Higer education	248 (91.51)	23 (8.49)	
**Place of residence**			
Village	129 (87.16)	19 (12.84)	0.5
City, with populations <50,000	77 (88.51)	10 (11.49)	
City, with populations 50,000–100,000	57 (82.61)	12 (17.39)	
City, with populations >100,000	272 (89.18)	33 (10.82)	

P_chi2_–ch^2^ test.

**Figure 1:**
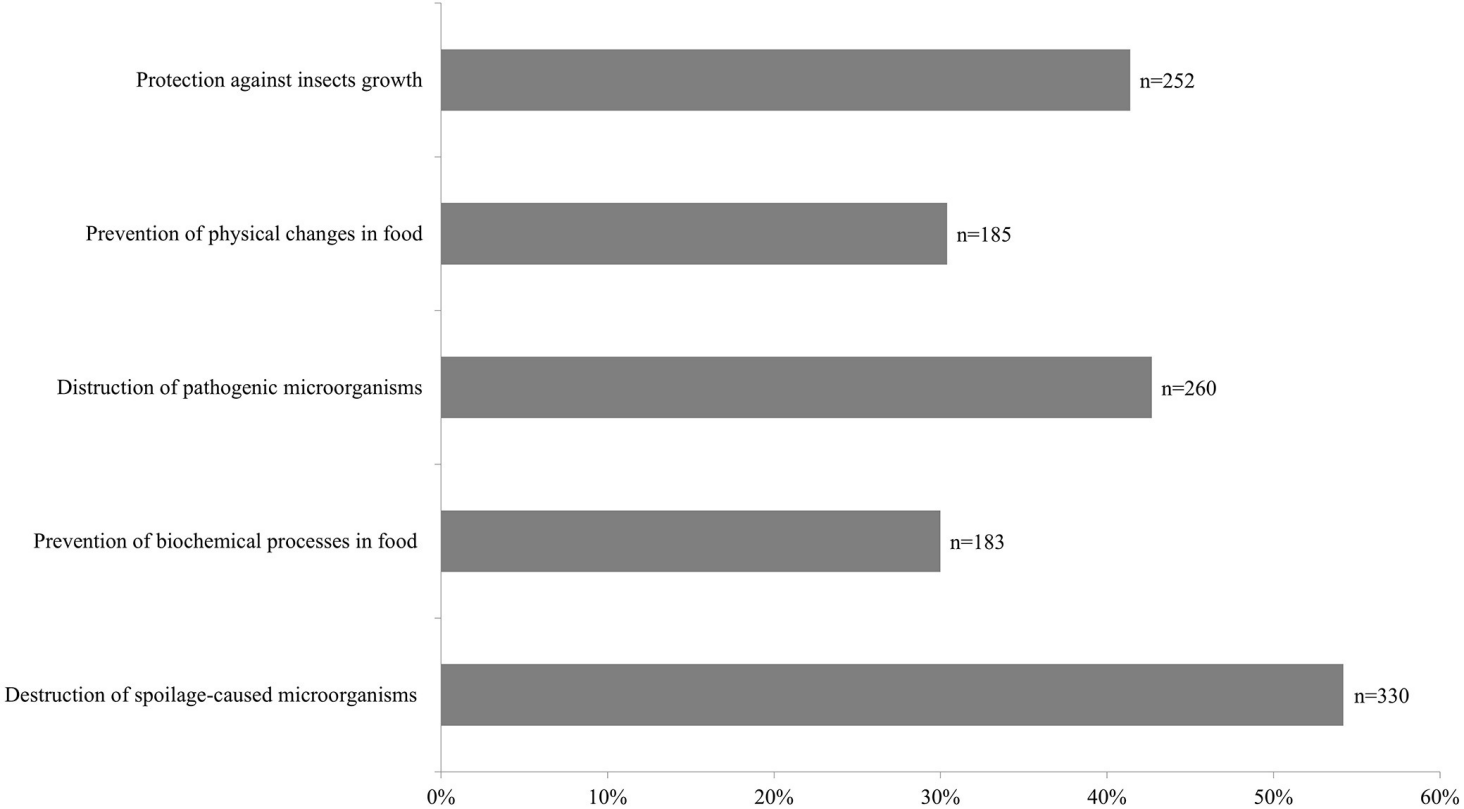
Factors that influence the choice of food preserved by the respondents (*multiple choice question*).

The most common factor encouraging the purchase of preserved products was the destruction of microorganisms can cause food spoilage (*n* = 330; 54.20%; Figure 1).

In the next stage of the study, respondents determined the frequency of consumption of food products subjected to different preservation methods. Based on the answers given, it can be assumed that, of the food groups listed, the most frequently consumed were “spices and dried vegetables”−46.00% (*n* = 280) and 32.80% (*n* = 200) of the respondents respectively declared that they consume this group of products, daily or several times a week. The analyses conducted also indicate the high popularity of items such as “UHT milk” and “packaged cheese and cured meats”; altogether, daily or several times a week, these products could be consumed by about 50% of the respondents. In contrast, the items most frequently chosen items were “canned fish” and “prepared vegetable salads”; more than half of the respondents indicated that they do not consume these products at all, or the frequency of their consumption is less than once a month.

When analyzing the relationship between the declaration of buying preserved foods and the frequency of consumption of products subjected to different preservation methods, it was observed that almost 70% of those who indicated that they did not buy preserved foods consumed “spices and dried vegetables” daily (*n* = 26; 35.10%) or almost daily (*n* = 25; 33.80%; *P*_chi2_ = 0.01) and 55.00% (*n* = 41) consumed “packaged cheese and cold cuts” at least once a week (*P*_chi2_ = 0.014).

### Respondents' attitudes toward irradiated food (before and after providing information materials)

The results obtained from the food irradiation section (before the educational materials) showed that up to 90.31% (*n* = 550) of the respondents had not previously heard of the use of ionizing radiation on food products. The question on the self-assessment of knowledge of irradiation shows that 58.10% (*n* = 354) of the respondents had an insufficient level of knowledge, while only 0.30% (*n* = 2) and 3.50% (*n* = 21) had a very good and good level of knowledge, respectively. 9.50% (*n* = 58) of the respondents felt that their knowledge of irradiation was sufficient, while 28.60% (*n* = 174) had no opinion on the subject. The low level of knowledge about the irradiation technique was reflected in the question in which the irradiated food symbol (Radura sign) had to be identified. Only 5.26% (*n* = 32) of the respondents gave the correct answer, that is, associated the label with irradiated products (*P*_M − W_ < 0.001).

**Figure 2:**
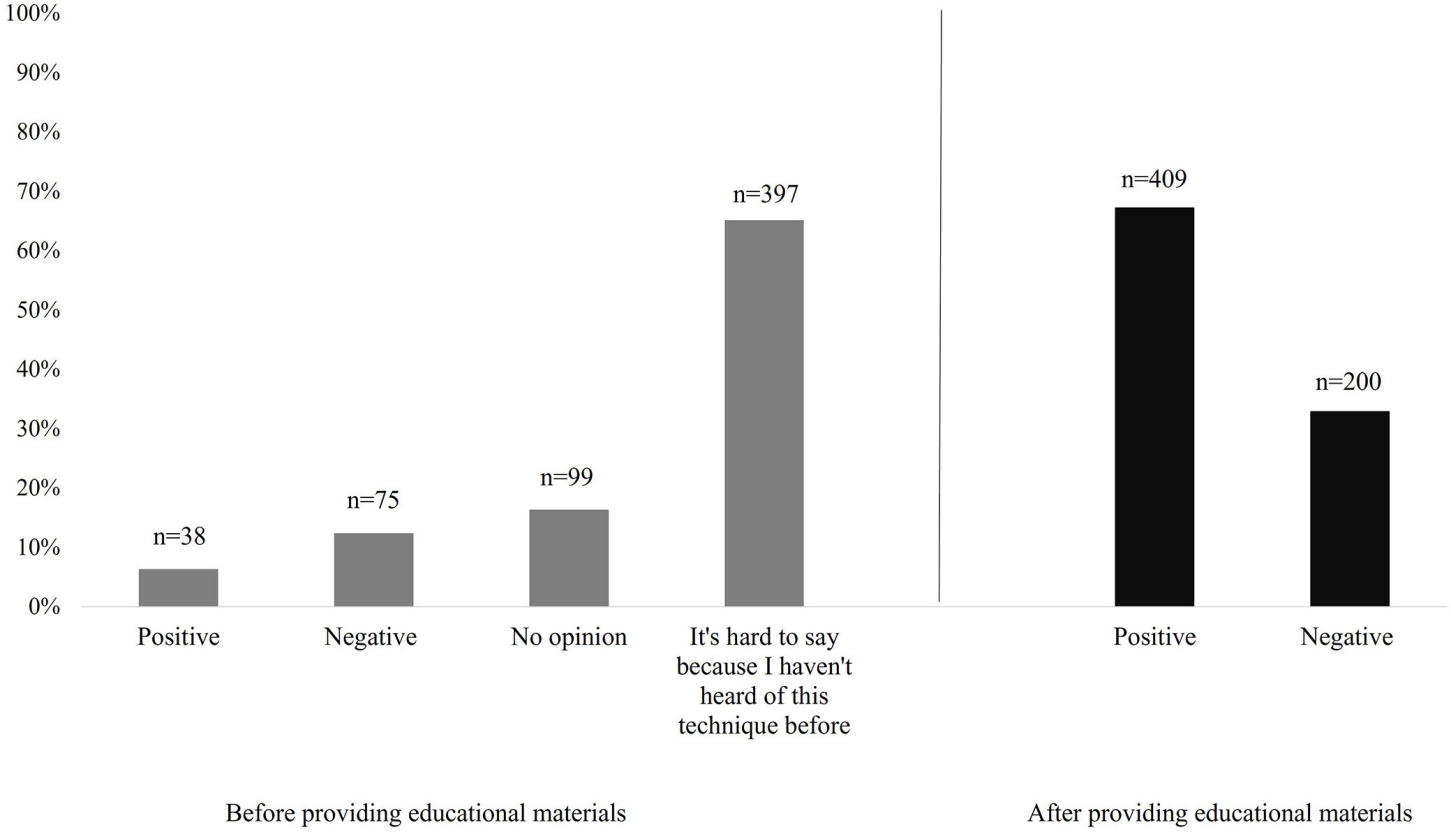
The attitudes of respondent toward food irradiation before and after providing educational materials.

The main objective of the survey conducted was to determine the attitude of the respondents toward food irradiation, before and after getting knowledge about the information material on this technique. At the beginning of the survey, the vast majority of participants, 81.46% (*n* = 496) could not specify their attitude toward this method, while only 6.20% (*n* = 38) of the respondents described their attitude as positive. However, after presentation, up to 67.16% (*n* = 409) of the respondents declared a positive attitude toward radiation-preserved food (Figure 2).

After providing informative material, about 70% of those who at the beginning of the survey could not determine their attitude toward irradiated food due to lack of knowledge on the subject described their attitude as positive. At the same time, about 35% of the respondents who initially assessed irradiated food negatively changed their opinion to positive (Table 3).

**Table 3:** Change in the attitudes of respondent toward food irradiation after providing educational materials.

**The attitudes of respondent toward food irradiation**			
**Before providing educational materials** ***n*** **(%)**	**After providing educational materials**		*P*^**B**^ **value**
	**Positive** ***n*** **(%)**	**Negative** ***n*** **(%)**	
Positive	37 (97.37)	1 (2.63)	0.001
Negative	26 (34.67)	49 (65.33)	
No opinion	65 (65.66)	34 (34.34)	
It's hard to say because I haven't heard of this technique before	281 (70.78)	116 (29.22)	

P^B^-Bhapkar test.

The attitudes of the respondents toward radiation-preserved food were also observed to be related to age, education, and place of residence, but only after the presentation of the information material. The percentage of respondents declaring positive attitudes toward irradiated products was highest among those aged 21–30 years (*n* = 269; 71.93%), those with higher education (*n* = 196; 72.32%) and those living in cities >100,000 inhabitants (*n* = 223; 73.11%; Table 4). There was no correlation between gender and respondents' attitudes toward radiation technology.

**Table 4:** The attitudes of respondent toward food irradiation after providing educational materials by sociodemographic factors.

	**The attitude toward food irradiation after providing educational materials**		***P*** **_chi2_ value**
	**Positive *n* (%) 409 (67.16)**	**Negative *n* (%) 200 (32.84)**	
**Gender**			
Female	299 (67.65)	143 (32.35)	>0.05
Male	110 (65.87)	57 (34.13)	
**Age group**			
18–20	87 (62.59)	52 (37.41)	0.008
21–30	269 (71.93)	105 (28.07)	
31–40	30 (62.50)	18 (37.50)	
41–50	17 (48.57)	18 (51.43)	
≥51	6 (46.15)	7 (53.85)	
**Educational level**			
Primary education	34 (54.84)	28 (45.16)	0.016
Secondary/professional education	179 (64.86)	97 (35.14)	
Higer education	196 (72.32)	75 (27.68)	
**Place of residence**			
Village	81 (54.73)	67 (45.27)	0.002
City, with populations < 50,000	58 (66.67)	29 (33.33)	
City, with populations 50,000–100,000	47 (68.12)	22 (31.88)	
City, with populations >100,000	223 (73.11)	82 (26.89)	

P_chi2_–ch^2^ test.

**Figure 3:**
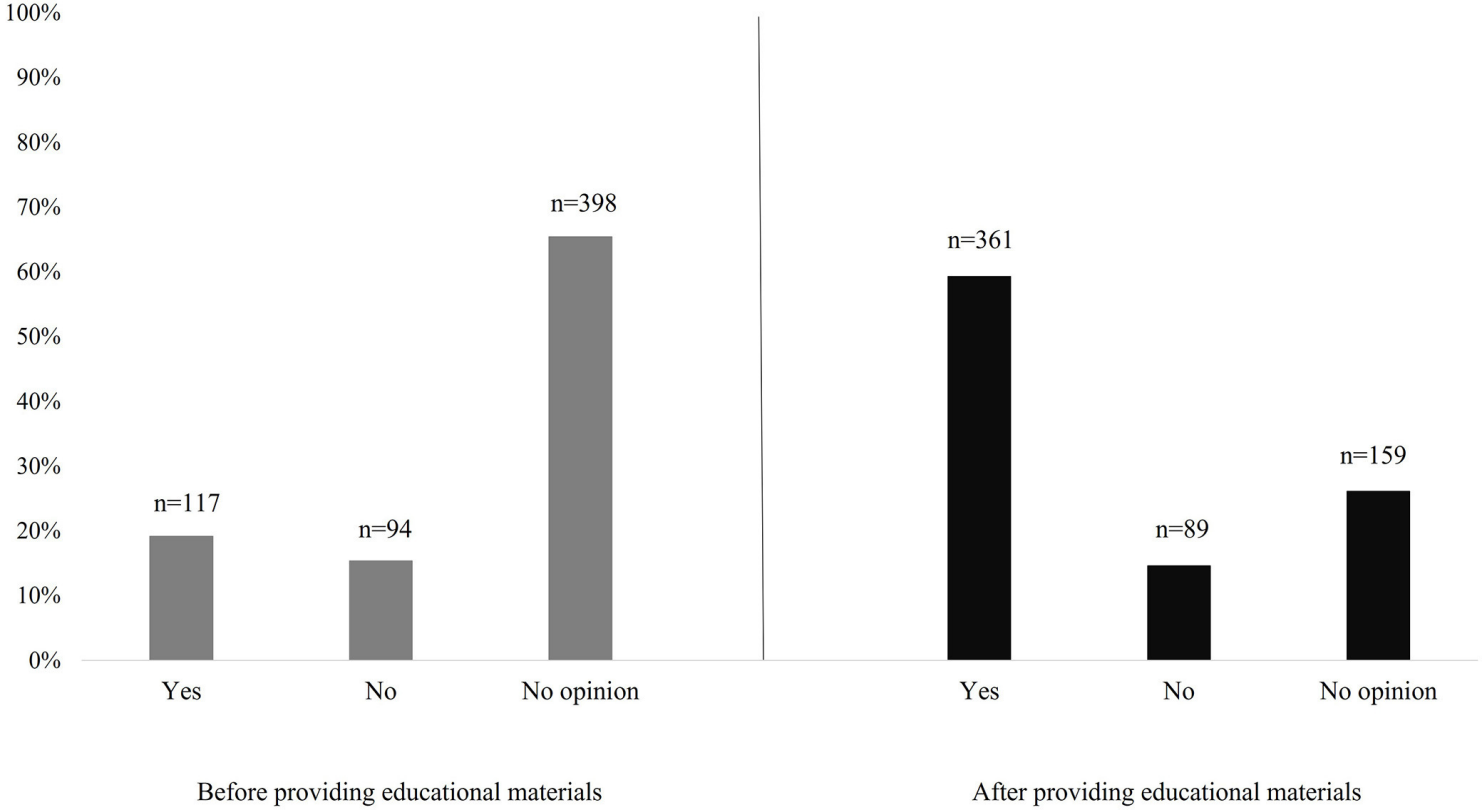
Irradiated food purchase declaration before and after providing educational materials.

Respondents' attitudes toward the possibility of purchasing radiation-preserved foods were also tested. Before providing educational materials, 19.20% (*n* = 117) of the participants declared their willingness to purchase such products, while after presentation, this number increased to almost 60% of the respondents (59.30%, *n* = 361; Figure 3).

It was observed that the presentation on food irradiation, had a significant effect on changing respondents' attitudes toward the possibility of purchasing radiation-preserved products. Almost 58% (*n* = 229) of the respondents, who initially had no opinion on the subject, expressed their willingness to purchase irradiated foods after the presentation (Table 5). At the same time, it was observed that the change in respondents' attitude toward the possibility of buying irradiated products depended on their general attitude toward food preservation—as many as 77.80% (*n* = 21) of those who declared that they do not buy preserved foods indicated, both before and after the presentation, that they would not choose to buy irradiated products either (*P*_F_ < 0.001).

**Table 5:** Change in the attitudes of respondent toward the possibility of purchasing radiation-preserved food after providing educational materials.

**Irradiated food purchase declaration**				
**Before providing educational materials**	**After providing educational materials**			***P*** **^W^-value**
	**Yes *n* (%)**	**No *n* (%)**	**No opinion *n* (%)**	
Yes *n*(%)	108 (92.31)	2 (1.71)	7 (5.98)	<0.001
No *n*(%)	24 (25.53)	56 (59.57)	14 (14.89)	
No opinion *n*(%)	229 (57.54)	31 (7.79)	138 (34.67)	

P^W^–Wilcoxon test.

The frequency of declaring the purchase of radiation-preserved products varied between women and men. Both before and after providing educational materials, women were, on average, more likely to be interested in purchasing irradiated foods—before presentation: women 19.90% (*n* = 88) vs. men 17.40% (*n* = 29; *P*_chi2_ < 0.05); after presentation: women 61.10% (*n* = 270) vs. men 54.50% (*n* = 91; *P*_chi2_ < 0.05). In contrast, other sociodemographic factors did not affect on the propensity to purchase irradiated products.

A weak positive correlation was found between self-assessment of knowledge and declaration of purchase of irradiated products (*V* = 0.23; *P* < 0.001)—respondents who could not assess their knowledge also had no opinion on the purchase of irradiated products.

**Figure 4:**
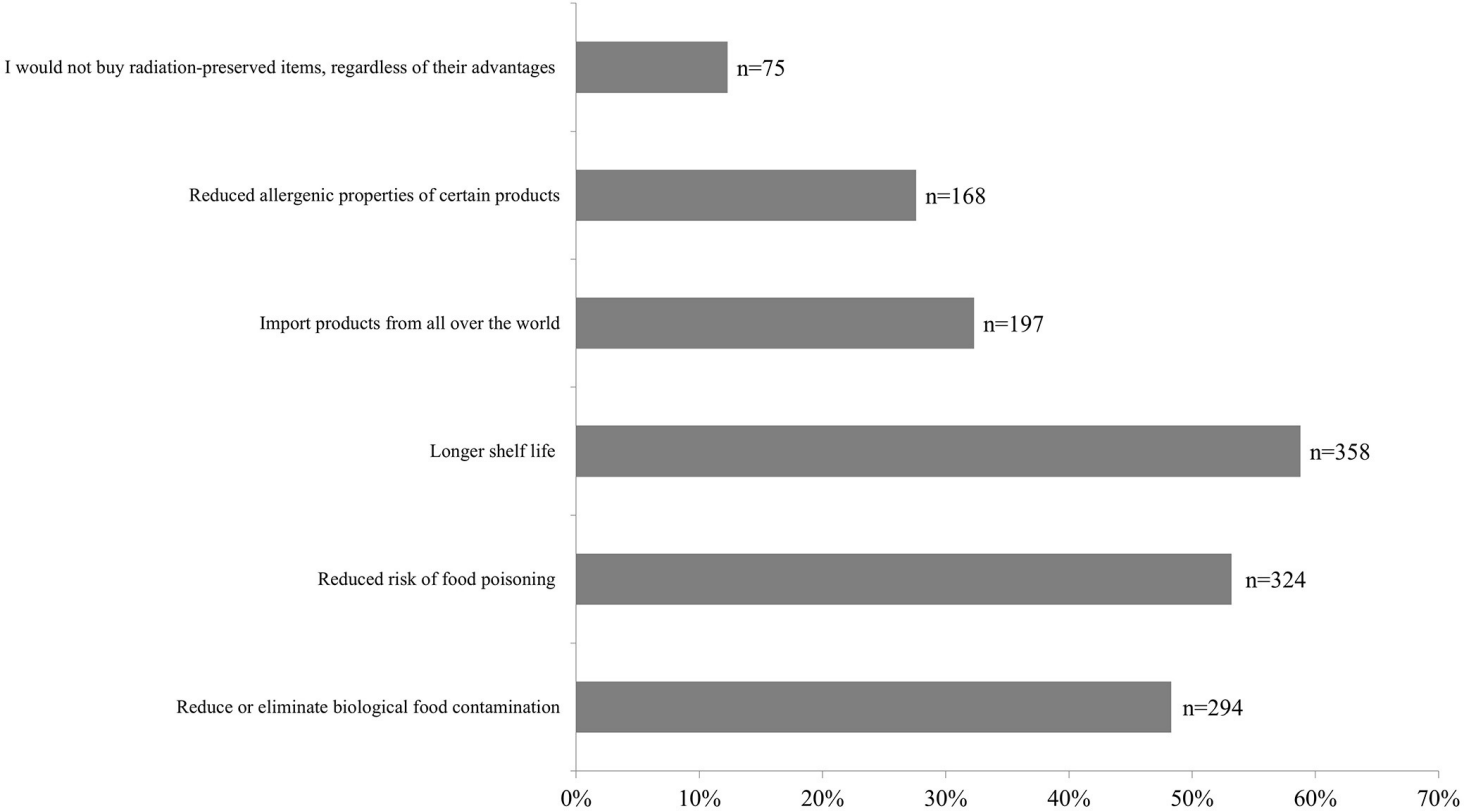
Advantages of the irradiation process that could be relevant when purchasing irradiated products: results after providing educational materials (*multiple choice question*).

The materials presented to respondents on food irradiation included information on the advantages of this method, among others. In the questionnaire part (only after familiarization with the materials), an attempt was made to determine which of the listed advantages might be most important to the respondents, when possibly purchasing this type of product. The most frequently indicated advantages of irradiated items were their longer shelf life (*n* = 358; 58.80%) and the reduced risk of food poisoning if consumed (*n* = 324; 53.20%). However, 12.30% (*n* = 75) of the survey participants indicated that they would not buy radiation-preserved items, regardless of their advantages (Figure 4).

## Discussion

Food irradiation is one of the food preservation methods currently used worldwide. The basis for the international acceptance of food irradiation was the decision of the Joint FAO/IAEA/WHO Expert Committee on the Wholesomeness of Irradiated Food (JECFI) in 1981, confirming the safety of products irradiated with doses below 10 kGy (17). In addition, in the 1990s, it was proven that nutrient losses accompanying the radiation process are low, especially at lower doses, and for those high doses (>10 kGy) can be similar to losses resulting from other food preservation methods, such as heat treatment (4).

Despite many opinions from national and international organizations and many scientific studies confirming the safety of the irradiation technique, the market for radiation-preserved foods has been slow to develop, which is particularly evident in European countries (9, 18). There may be several reasons for this phenomenon, but the lack of consumer knowledge about food safety in the broadest sense seems to play a key role. It has been observed that consumers have a misconception about the food processing and preservation techniques used, they are more concerned about the presence of food additives and ingredients derived from genetic modification in food products than about microbiological contamination (19). The observed phenomenon may have serious health consequences, especially since the number of cases of diseases resulting from the consumption of contaminated food remains high. In the US, the estimated annual number of cases of foodborne illness is 47.8 million, while in the European Union, 20,017 cases of such illnesses were reported in 2020 (20, 21). This is also compounded by trends and food preferences among consumers. In the last decade or so, there has been a marked increase in consumer concern in developed countries about new, innovative methods of food production and processing, with the belief that the 'traditional appearance' of a product guarantees its superior quality. As a result, some consumers treat unprocessed products as always healthy and safe, forgetting that food can be contaminated at any stage of the food chain, including production, storage, or food preparation (22, 23). Current dietary trends are reflected in Centers for Disease Control and Prevention (CDC) and EFSA data—among the main causes of food poisoning and other foodborne illnesses, fresh products such as raw meat, fruit and vegetables, plant sprouts or eggs predominate (20, 24). The medical costs of food-related illnesses have been estimated at US$ 6.5–34.9 billion, while recalls of contaminated products and loss of consumer confidence cost the food sector around US$ 39 billion per year. At the same time, irradiation, among various antimicrobial sanitization methods, has been identified as one of the most effective interventions, outperforming chemical agents and newly developed non-thermal methods (25).

Despite the widely observed decline in confidence in various food preservation methods, in this study, up to 90% of respondents declared that they buy preserved products, most often (around 57%) due to the reduced spoilage of such foods. This high percentage of people buying preserved products may be due to the specific period in which the survey was conducted, the COVID-19 pandemic. In many countries, a significant proportion of the population has been shown to change their eating and shopping habits during the pandemic. The increased consumption of products with long shelf life was observed, while the consumption of fresh items declined. The reasons for this phenomenon can be attributed, firstly, to less frequent grocery shopping due to the epidemic threat and the restrictions put in place, and secondly, to a decrease in household financial income (preserved products are generally cheaper than fresh ones) (26–28).

The propensity to buy canned products may also be due to the age structure of the participants in this study—around 80% of the respondents were adults under the age of 30, who are generally more positive about new technologies. The high proportion of young participants in the survey is probably due to how the questionnaires are delivered—as other research reports indicate, when on-line surveys are used, there is a tendency for the age of participants to be lower, as younger people are more engaged and spend more time online (29).

This study also showed that preserved food was more likely to be purchased by women. It seems that women pay more attention to food safety and are more aware of the risks associated with it (30). Dai et al. (31) showed that women were 22% more likely to prefer safe food products compared to men. Women's higher declaration of buying preserved products may also be influenced by the fact that grocery shopping is more frequent among this group, due to the culturally determined greater responsibility for feeding the family and managing the household (32).

Food irradiation as one of the preservation methods is not very widespread among Polish consumers—only 10% of the participants in this study knew about the possibility of applying ionizing radiation to food, and only 4% of them rated their knowledge at a very good or good level. It appears that Polish consumers are unaware of the preservation of food radiation. Research conducted in the 1990s in the USA showed that the concept of food irradiation was known to 48%−72% of the participants (33, 34), while in Canada the percentage was 43% (35). Less awareness of radiation technology was observed in South America (in Chile, only 23.5% of the respondents declared knowledge of the irradiation process) and in Europe (29% of Turkish consumers knew about the possibility of using ionizing radiation in the food industry) (36, 37).

The low level of familiarity with the irradiation technique observed in this study translates into the attitudes of the respondents toward radiation-fixed food, which was positively rated by only 6% of the participants. Very similar results were obtained in a 2016 study that looked at consumer attitudes toward various food preservation solutions—only 6.6% of Polish respondents considered irradiated food safe (38). This is a direct consequence of a lack of knowledge about the irradiation process and fear of ionizing radiation. Scientific reports indicate that the term irradiation often evokes negative consumer associations with nuclear disasters or cell destruction. In addition, many times consumers believe that irradiated products become radioactive, pose a threat to the environment, and have lower nutrient content, which is contrary to scientific reports (36, 39). Bolek (19) and Ergönül (40) noted that irradiated foods were rated as dangerous or extremely dangerous by 70%−76% of respondents. Similar results were obtained by Gunes and Tekin (37), with only 11% of respondents rating radiation-fixed foods as safe. Europeans are generally more distrustful of irradiated food than American, Chinese or Korean consumers (41).

Consumer education is crucial for the acceptance of new technologies, as is evident in this study—after the presentation of materials containing basic information about the irradiation process, the percentage of respondents who viewed the technique positively increased by more than 60%, at 67.16%. It is noteworthy that 35% of respondents who initially evaluated irradiation negatively after the presentation described their attitude as positive.

Scientific reports indicate that educational programs contribute to a significant increase in public awareness and translate into greater acceptance of food irradiation. The more consumers know about the technique, the more willing they are to use it. Even a minimal amount of information can lead to a significant increase in acceptance (42). Lack of adequate knowledge is a major factor limiting the wider use of radiation technology in the food industry (36). A study in Turkey showed that thanks to educational materials, positive attitudes toward irradiated foods increased among participants from 29 to 66%, and 62% of respondents declared a willingness to purchase these products (37). On the contrary, Galati et al. (43) noted that 84.2% of Italian respondents were not familiar with the method of preserving food using ionizing radiation, but at the same time, 89.2% of respondents were interested in receiving information on the subject. In Argentina, the provision of information on food irradiation resulted in a 90% increase in acceptance of this technique (44). The positive effect of information materials on the perception and decision to purchase irradiated food was also confirmed by Oliveira and Sabato (45), Nayga et al. (46), Behrens et al. (47). Buyn et al. (48), on the other hand, showed that attitudes toward irradiated food were also significantly influenced by how individual information was presented: the group that listened to an expert lecture represented the highest level of positive attitudes toward irradiated food, compared to groups that received information in video or text form.

All information activities should be linked to the availability of the selected items on the market, which allows the consumer to make an initial assessment of the quality of such a product. Deliza et al. (49) observed that the appearance of a product is the most important factor influencing the decision to buy it. Importantly, the price and the presence of information that the article has been irradiated are less important. Unfortunately, irradiated articles are hard to be found on the Polish market, and the only unit in Poland authorized to irradiate food uses irradiation only for scientific purposes (9). The best example of how a properly conducted educational campaign can influence consumer attitudes toward radiation-fixed products is the United States. Educational activities in this country, combined with the sale of irradiated products, resulted in the percentage of people willing to purchase radiation-fixed foods increasing from 29% in 1993 to 69% in 2003 (50, 51).

Consumers' attitudes toward the radiation technique may depend not only on the level of knowledge but also on selected socio-demographic factors. In the present study, following the presentation of educational materials, it was observed that respondents' attitudes toward the irradiation process were significantly related to age, level of education, and place of residence. The proportion of respondents with a positive attitude toward the technique was highest among the youngest respondents (<30 years of age) and increased with elevated educational level and level of urbanization. The results obtained can be related to studies on food and technology neophobia, i.e., aversion to trying new foods and aversion to new food processing technologies. Siegrist et al. (52) found that food neophobia correlates positively with age and negatively with education level and urbanization. Similar findings were found by Vidigal et al. (53)—a greater propensity for technological neophobia is found among those aged >36 years and the poorly educated. The effect of gender on consumer attitudes toward food irradiation was not identified in the present study—on the one hand, there were no differences between men and women in the proportion of people with a positive attitude toward radiation, but on the other hand, women were significantly more interested in purchasing irradiated food compared to men (61.10 vs. 54.50%). The relationship between neophobia and gender has also not been resolved. Siegrist et al. (52) indicate that men have higher levels of neophobia than women, which may be due to cultural factors, while other studies suggest no relationship between these variables (52, 54). Based on our results and other scientific reports, it can be assumed that an older consumer with little access to information (residents of small towns) and poor education is unlikely to be interested in purchasing “new, unfamiliar” foods, including irradiated foods (55).

The study conducted also has some limitations. Due to the significant participation of people under 30 years of age in the survey, the generalizability of the results to the entire population may be questionable. In addition, the information material presented to respondents focused primarily on the advantages of the irradiation technique, including the risks that can be eliminated through its use. In addition, the inclusion of information about possible limitations and some disadvantages of the process could have changed the final attitude of the respondents, so future research should focus on checking the potential relevance of such data.

Food irradiation is undoubtedly an effective and safe food preservation technique that, when used alone or in combination with other preservation methods, can help national and international producers and suppliers provide consumers with the safest and highest quality food products possible. Furthermore, in an era of climate change that affects food availability, greater use of irradiation can have a measurable impact on reduced food waste. Unfortunately, without appropriate educational campaigns, it will not be possible to spread irradiated products in Poland, due to the low level of consumer knowledge and awareness in this area. The survey conducted, which is the first in Poland and one of the few in Europe, can help to plan an effective educational program, aimed primarily at young and educated people, as they are the most open to new technologies.

## Data availability statement

The original contributions presented in the study are included in the article/supplementary material, further inquiries can be directed to the corresponding author.

## Ethics statement

Ethical review and approval was not required for the study on human participants in accordance with the local legislation and institutional requirements. Written informed consent for participation was not required for this study in accordance with the national legislation and the institutional requirements.

## Author contributions

MB and MG contributed in data analysis and interpretation and wrote the article. MB approved the final version of article. AD and DS contributed to the research concept and design. AD, DS, and AK contributed to the data collection and collation. MG contributed to the translation of the article. JM-B and AS wrote and translated the article. All authors contributed to the article and approved the submitted version.
